# Estimating the real-world effects of expanding antiretroviral treatment eligibility: Evidence from a regression discontinuity analysis in Zambia

**DOI:** 10.1371/journal.pmed.1002574

**Published:** 2018-06-05

**Authors:** Aaloke Mody, Izukanji Sikazwe, Nancy L. Czaicki, Mwanza Wa Mwanza, Theodora Savory, Kombatende Sikombe, Laura K. Beres, Paul Somwe, Monika Roy, Jake M. Pry, Nancy Padian, Carolyn Bolton-Moore, Charles B. Holmes, Elvin H. Geng

**Affiliations:** 1 Division of HIV, ID and Global Medicine, University of California, San Francisco, Zuckerberg San Francisco General Hospital, San Francisco, California, United States of America; 2 Centre for Infectious Disease Research in Zambia, Lusaka, Zambia; 3 Department of International Health, Johns Hopkins University School of Public Health, Baltimore, Maryland, United States of America; 4 Department of Public Health, University of California, Davis, Davis, California, United States of America; 5 Division of Epidemiology, University of California, Berkeley, Berkeley, California, United States of America; 6 Division of Infectious Diseases, University of Alabama, Birmingham, Alabama, United States of America; 7 Division of Infectious Diseases, Johns Hopkins University School of Medicine, Baltimore, Maryland, United States of America; University of Southampton, UNITED KINGDOM

## Abstract

**Background:**

Although randomized trials have established the clinical efficacy of treating all persons living with HIV (PLWHs), expanding treatment eligibility in the real world may have additional behavioral effects (e.g., changes in retention) or lead to unintended consequences (e.g., crowding out sicker patients owing to increased patient volume). Using a regression discontinuity design, we sought to assess the effects of a previous change to Zambia’s HIV treatment guidelines increasing the threshold for treatment eligibility from 350 to 500 cells/μL to anticipate effects of current global efforts to treat all PLWHs.

**Methods and findings:**

We analyzed antiretroviral therapy (ART)-naïve adults who newly enrolled in HIV care in a network of 64 clinics operated by the Zambian Ministry of Health and supported by the Centre for Infectious Disease Research in Zambia (CIDRZ). Patients were restricted to those enrolling in a narrow window around the April 1, 2014 change to Zambian HIV treatment guidelines that raised the CD4 threshold for treatment from 350 to 500 cells/μL (i.e., August 1, 2013, to November 1, 2014). Clinical and sociodemographic data were obtained from an electronic medical record system used in routine care. We used a regression discontinuity design to estimate the effects of this change in treatment eligibility on ART initiation within 3 months of enrollment, retention in care at 6 months (defined as clinic attendance between 3 and 9 months after enrollment), and a composite of both ART initiation by 3 months and retention in care at 6 months in all new enrollees. We also performed an instrumental variable (IV) analysis to quantify the effect of actually initiating ART because of this guideline change on retention. Overall, 34,857 ART-naïve patients (39.1% male, median age 34 years [IQR 28–41], median CD4 268 cells/μL [IQR 134–430]) newly enrolled in HIV care during this period; 23,036 were analyzed after excluding patients around the threshold to allow for clinic-to-clinic variations in actual guideline uptake. In all newly enrolling patients, expanding the CD4 threshold for treatment from 350 to 500 cells/μL was associated with a 13.6% absolute increase in ART initiation within 3 months of enrollment (95% CI, 11.1%–16.2%), a 4.1% absolute increase in retention at 6 months (95% CI, 1.6%–6.7%), and a 10.8% absolute increase in the percentage of patients who initiated ART by 3 months and were retained at six months (95% CI, 8.1%–13.5%). These effects were greatest in patients who would have become newly eligible for ART with the change in guidelines: a 43.7% increase in ART initiation by 3 months (95% CI, 37.5%–49.9%), 13.6% increase in retention at six months (95% CI, 7.3%–20.0%), and a 35.5% increase in the percentage of patients on ART at 3 months and still in care at 6 months [95% CI, 29.2%–41.9%). We did not observe decreases in ART initiation or retention in patients not directly targeted by the guideline change. An IV analysis found that initiating ART in response to the guideline change led to a 37.9% (95% CI, 28.8%–46.9%) absolute increase in retention in care. Limitations of this study include uncertain generalizability under newer models of care, lack of laboratory data (e.g., viral load), inability to account for earlier stages in the HIV care cascade (e.g., HIV testing and linkage), and potential for misclassification of eligibility status or outcome.

**Conclusions:**

In this study, guidelines raising the CD4 threshold for treatment from 350 to 500 cells/μL were associated with a rapid rise in ART initiation as well as enhanced retention among newly treatment-eligible patients, without negatively impacting patients with lower CD4 levels. These data suggest that health systems in Zambia and other high-prevalence settings could substantially enhance engagement even among those with high CD4 levels (i.e., above 500 cells/μL) by expanding treatment without undermining existing care standards.

## Abstract 2

In a regression discontinuity analysis, Aaloke Mody and colleagues assess the implications for treatment initiation and retention of expanded eligibility for antiretroviral therapy in Zambia.

## Author summary

**Why was this study done?:**

- Universal treatment with ART regardless of CD4 count (i.e., treat-all) is based on randomized controlled trials showing the biologic efficacy of ART, but the public impact of expanding ART eligibility also depends on the behavior of patients and the capacity of health systems to absorb an influx of new patients.
- Direct effects of expanding ART eligibility include increased ART initiation, but it may also indirectly affect patient behavior (i.e., retention) or create negative spillover effects through increased clinic congestion and crowding out of patients already eligible for ART.
- We leveraged a 2014 change to Zambia’s national ART guidelines that expanded ART eligibility to those with a CD4 between 350 and 500 cells/μL and pregnant or breastfeeding women to anticipate the effects of expanding treatment to those with CD4 above 500 cells/μL under treat-all.

**What did the researchers do and find?:**

- We used a regression discontinuity design to compare patients enrolling just before and after this 2014 change to Zambia’s national ART guidelines expanding the CD4 treatment threshold from 350 to 500 cells/μL to estimate the effects of the guideline change on ART initiation and retention in care in the entire patient population.
- Among 34,587 patients who newly enrolled in HIV care between August 1, 2013, and November 1, 2014, Zambia’s 2014 guideline change was associated with a 13.6% increase in the proportion initiating treatment within 3 months of enrollment, a 4.1% increase in retention in care at 6 months, and a 10.8% increase in the percentage of patients in care and on ART at 6 months in the entire clinic population.
- These effects were most pronounced in those patients who would have become newly eligible with the guideline, but ART initiation also increased without evidence of decreased retention in those who remained eligible as well as those who were not yet eligible for ART.
- We estimated that initiating ART in response to the change in guidelines led to a 37.9% increase in retention in care, indicating that 2.6 patients would need to be initiated on ART to prevent one episode of LTFU.

**What do these findings mean?:**

- Expanding ART eligibility to patients with higher CD4 counts in a real-world setting was associated with marked changes in patient behavior that were not observed in the more tightly controlled settings of randomized trials.
- Expanding treatment eligibility was associated with modest increases in ART initiation, overall retention in care, and the percentage of patients in care and on ART, without increasing clinic congestion or negatively impacting those who were already eligible for treatment before the 2014 guideline change (i.e., those with a CD4 less than 350 cells/μL at the time of enrollment).
- Adopting treat-all may lead to similar improvements in ART initiation and retention without associated negative effects; further research regarding the real-world effects of implementing treat-all is needed.
- Although expanding treatment eligibility improved ART initiation and retention in care, the overall HIV care cascade remained suboptimal. Further innovations in health systems are needed to increase early diagnosis and linkage and improve overall ART initiation and retention in care.

## Introduction

Although randomized controlled trials (RCTs) have demonstrated that antiretroviral therapy (ART) in persons living with HIV (PLWHs), irrespective of CD4 levels, has broad clinical benefits, policies to expand HIV eligibility in the real world may have important additional effects that are not captured in the context of controlled trials. HPTN 052 first demonstrated in 2010 that treating the positive partner in a serodiscordant relationship reduces HIV transmission by as much as 96%. This was followed in 2015 by the INSIGHT START and TEMPRANO trials, which showed that immediate treatment reduced the incidence of serious AIDS-related events by 50%, even in patients with CD4 levels greater than 500 cells/μL [1–3]. Based on these trials, the World Health Organization (WHO) in 2015 recommended treatment for all individuals infected with HIV, irrespective of CD4 levels, and, over the last 2 years, national governments in high-prevalence regions are gradually adopting the treat-all approach as a cornerstone of HIV control programs [4,5]. Nevertheless, the public health impact of adopting a treat-all policy is still unknown, because it depends on the capacity of health systems to engage and absorb a new population as well as the behavior of these patients in routine healthcare settings [6].

The current clinical evidence base for offering treatment to all persons with HIV provides limited insights on its anticipated effects in routine care settings. Broadly speaking, implementation science is motivated by the observation that guidelines rarely result in all intended effects and often lead to at least some unintended effects. Typically, individual-level RCTs establish clinical efficacy by only enrolling participants already willing to be treated, closely monitoring both treated and untreated patients, and delivering care in regulated environments to confidently capture the biological effect of treatment [7–9]. In INSIGHT START and TEMPRANO, for example, the tightly regulated care setting led to close to 100% of treatment-eligible patients initiating ART and greater than 95% of patients retained in both the immediate-ART and deferred-ART arms [2,3]; yet, in real-world, routine-care settings, offering treatment to persons with higher CD4 levels may engage them in the system and thereby improve retention. Alternatively, increasing treatment may reduce attention to those with the lowest CD4 levels who were already eligible before the policy change, potentially adversely affecting initiation and retention. Intriguing work from South Africa found that offering treatment to patients with a CD4 just below 350 cells/μL (compared to those just above) led to improved retention [10]. This study, however, did not directly speak to unintended health systems consequences of expanding treatment eligibility, such as clinic congestion, decreased quality of care, and crowding out of sicker patients who were already eligible for treatment across a wider range of CD4 levels [6,11–19].

On April 1, 2014, Zambia updated its HIV treatment guidelines to expand ART eligibility to asymptomatic patients by increasing the CD4 threshold for treatment from 350 to 500 cells/μL and instituting treatment for all pregnant and breastfeeding women under Option B+ [20,21]. We use this guideline change to estimate the effect of expanding eligibility on the behavior of patients (e.g., retention) and health systems (e.g., crowding out) through regression discontinuity and instrumental variable (IV) analyses. In this approach, we treat patients who present immediately before and after guideline implementation as “exchangeable” and therefore interpret differences at the cutoff plausibly as the effect of the guideline change. In addition, we use exposure to the new guidelines as an IV to estimate the effect of actually initiating ART because of the guideline change on retention in care. Overall, we seek to provide evidence that will help anticipate the effects of ongoing worldwide efforts to expand treatment eligibility to all PLWHs.

## Methods

### Ethics statement

The study was approved by the University of Zambia Biomedical Research Ethics Committee (UNZABREC) and institutional review boards at the University of California, San Francisco, and the University of Alabama, Birmingham, School of Medicine. The research in this paper was not prespecified in the original study protocol and consists of secondary analyses that were conceptualized to take advantage of the change to Zambian national guidelines that occurred during the study period. This manuscript was prepared according to STROBE guidelines (S1 STROBE Checklist).

### Patient population and setting

We analyzed ART-naïve, HIV-infected adults (greater than 15 years old) who newly enrolled in HIV care before and after the April 1, 2014 change in Zambia’s HIV treatment guidelines, from August 1, 2013, to November 1, 2014. Patients were from one of 64 clinics operated by the Zambian Ministry of Health that received technical support from the Centre for Infectious Disease Research in Zambia (CIDRZ), a Zambian nongovernmental organization that supports implementation of HIV care delivery and research across 4 of the 10 provinces in Zambia. According to Zambian national guidelines at the time of this study, patients eligible for ART first underwent three evaluation and counselling sessions prior to being initiated on ART about 3–6 weeks later. They were then followed up monthly for at least the first 6 months of treatment, after which they might be eligible to have their visits spaced out to 3-month intervals if considered stable. Patients ineligible for ART at the time of enrollment were followed up with clinical evaluations and CD4 counts every 6 months until becoming eligible for ART.

### Measurements

Measurements were extracted from the national electronic medical record system used in routine HIV care in Zambia, SmartCare. This system involves providers manually filling out paper clinical forms during patient encounters in routine practice with data clerks, then entering this information into the electronic database. We used patient sociodemographic characteristics (e.g., age, sex, clinic site), clinical characteristics (enrollment CD4 count, WHO stage, TB diagnoses, pregnancy, breastfeeding), and visit history (enrollment date, date of ART initiation, follow-up visits) for our analyses.

### Analysis

#### Descriptive analysis of time to ART initiation and loss to follow-up

First, we describe in a multistate analysis the longitudinal experience of patients enrolling in HIV care, stratified by pre- and post-guideline change, by estimating the probability of a patient being in a particular care state at a given time point after enrollment. We defined four mutually exclusive and exhaustive states: (1) in care and not initiated on ART, (2) in care and on ART, (3) lost to follow-up (LTFU; defined as being greater than 90 days late to the next scheduled appointment or 180 days since any visit, if no scheduled visit recorded) or died prior to initiating ART, and (4) LTFU or died after initiating ART. Death was combined with LTFU because of known inaccuracies in ascertainment of mortality from routine programmatic data [22]. We then performed a multistate analysis using a modified approach to the Aalen-Johansen method for competing risks to account for the fact that patients may transition through multiple retention states over time [23,24]. In this approach, we performed separate analyses for each time point to obtain estimates for that interval, thereby allowing patient statuses to transition through nonabsorbable states and be updated over time. This approach, for example, accounts for a patient who becomes LTFU but then re-enters care at a later time. For each analysis, time zero was considered the day of enrollment and patients were censored at the date of their most recent event. Patients with no event were administratively censored at the time of their last visit, date of transfer, or—for those patients who enrolled prior to the new guidelines—at the time of guideline rollout. In this way, the prevalence of a number of patient conditions (e.g., on ART, retained, lost) can be estimated at any given time after enrollment, when observation time across the population is unequal.

#### Effect of change in guidelines on ART initiation and retention in care using regression discontinuity

Second, we used a regression discontinuity design to estimate the effect of the change in treatment eligibility criteria on three outcomes in the entire population enrolling in care: (1) timely ART initiation (defined as within 3 months of enrollment), (2) retention in care at 6 months (defined as having made at least one visit between 3 and 9 months post-enrollment), and (3) being retained and on ART at 6 months (a composite outcome of having timely ART initiation and being in care at 6 months). The regression discontinuity approach potentially permits causal inferences by comparing patients just above or below an arbitrary threshold value in a small window or bandwidth around the threshold (i.e., “locally”). This approach makes the assumption that patients immediately on either side will be similar on both observed and unobserved characteristics (i.e., “exchangeable”), thus making assignment to exposure “as if” random [25–27]. In this analysis, we used the date of new guideline issuance (April 1, 2014) as the threshold value and compared outcomes in those patients enrolling in care before and after that cutoff to estimate the effect of the guideline change. All patients were followed for at least 9 months in order to have full outcome ascertainment.

Statistically, we estimated the effects of expanding ART eligibility by modelling a modified Poisson regression with robust variances—including an interaction between guideline change and time to allow for a change in slope and intercept at the threshold—and estimating the risk difference (RD) for each outcome at the time of guideline rollout [26–29]. To avoid bias from patients enrolling pre-guidelines “crossing over” and having outcomes (of ART initiation and retention) influenced by exposure to practices both before and after the guideline change, we restricted our analysis to patients enrolling at least 90 days prior to implementation of the guidelines. We also restricted it to those enrolling at least 60 days after implementation to account for a limited transition period during the rollout of guidelines. We included the full bandwidth (i.e., the window around the cutoff to be analyzed) of data in our final models to avoid identifying artifactual trends that were due to short-term random variability and were without substantive meaning. Our final models were unadjusted based on the assumption of exchangeability, and we weighted observations equally within this window using a rectangular kernel. We assessed for violations of the underlying assumptions by (1) comparing the characteristics of patients enrolling before and after the guidelines using a *t* test or chi-squared test, as appropriate, (2) graphically confirming that there were no gross aberrations in the trends of new enrollees and overall clinic visits per week to suggest changes in other external factors that could affect patient behaviors, and (3) formally testing for continuity in the density of new enrollees at the threshold using the McCrary test (*p* = 0.86 for discontinuity at the threshold) [30].

As regression discontinuity analyses can be sensitive to choices in model specification, we conducted multiple sensitivity analyses to ensure that our results remained robust under different model specifications. First, we assessed different bandwidths around the threshold value—including using Imbens-Kalyanaram data-driven bandwidths—to ensure that results remained robust irrespective of the chosen bandwidth [25,27]. The Imbens-Kalyanaram data-driven algorithm attempts to objectively identify the largest window around the cutoff in which the relationship between time and the outcome are approximately linear, thus attempting to maximize precision but minimize bias from nonlinearity of this relationship further away from the cutoff [31]. Second, we assessed a shorter 30-day transition period for the guideline rollout (as opposed to 60 days). Third, we confirmed results were unchanged when adjusting for sex, age, CD4 count at enrollment, and clinic to assess for sensitivity to any changes in the composition of newly enrolling patients before and after the change in guidelines. Lastly, we used a triangular-shaped kernel to weight patients proportional to how close they enrolled to guideline implementation, assigning greatest weight to those enrolling closest to implementation, to assess whether results were driven by those furthest from the threshold [25,28].

#### Patient eligibility subgroups

In patients with complete eligibility information, we defined three patient eligibility subgroups based on patients’ clinical characteristics at enrollment but regardless of their actual date of enrollment to identify potential spillover effects in groups not targeted by the change in guidelines. We classified “always eligible” patients as those who would have been eligible for ART initiation by the 2010 guidelines at enrollment (CD4 count less than 350 cells/μL, WHO clinical stage 3 or 4, or active tuberculosis), “newly eligible” patients as those who would have been eligible for ART initiation by the 2014 guidelines but not according to the 2010 guidelines (enrollment CD4 count between 350 and 500 cells/μL, pregnant or breastfeeding women), and “not yet eligible” patients as those who would not have been considered eligible for ART initiation by either the 2010 or 2014 guidelines (enrollment CD4 count greater than 500 cells/μL, with no other qualifying criteria). “Newly eligible” patients were further stratified into those newly eligible because of CD4 count alone or Option B+. Because of data limitations, we were unable to ascertain patients with serodiscordant partners or hepatitis B virus (HBV) coinfection with severe liver disease, who would also have been eligible by the 2010 guidelines, or partners of pregnant or breastfeeding women, who would have been newly eligible with the 2014 guidelines [20,21].

#### Effect of initiating ART on retention in care using guideline exposure as an IV

Lastly, we sought to assess how actually initiating ART in routine care affects retention in care through an IV analysis. Due to the observational nature of our data, it is likely that there are unobserved common causes of ART initiation (e.g., the “treatment” in this analysis) and retention (e.g., the outcome) that will confound the relationship using traditional regression methods. An IV approach—widely used in econometrics and increasingly in public health—can overcome this unmeasured confounding under certain assumptions that are plausible in this setting: (1) the IV (e.g., exposure to guidelines in this case) is associated with treatment (e.g., ART initiation), (2) the IV is not associated with the outcome (e.g., retention) except through its relationship with the treatment, (3) there are no common causes (i.e., unmeasured confounders) of the IV and the outcome, and (4) exposure to the IV does not preclude treatment in those who would have otherwise been treated [25,32,33]. Under the aforementioned conditions, this method estimates the causal effect of ART initiation on retention in care in those patients who initiated ART in response to the change in guidelines—commonly referred to as the local average treatment effect (LATE) [25,32,33].

Statistically, we used a two-stage least squares bivariate probit regression. During the first stage, we modelled the relationship between exposure to the guidelines and ART initiation and then used the predicted values of ART initiation in the second stage to estimate the association with retention. Patient restriction was equivalent to that used in the regression discontinuity analysis. Prior to conducting the IV analysis, we empirically tested for violations of the underlying IV assumptions (S1 Appendix).

## Results

### Patient characteristics

**Fig 1:**
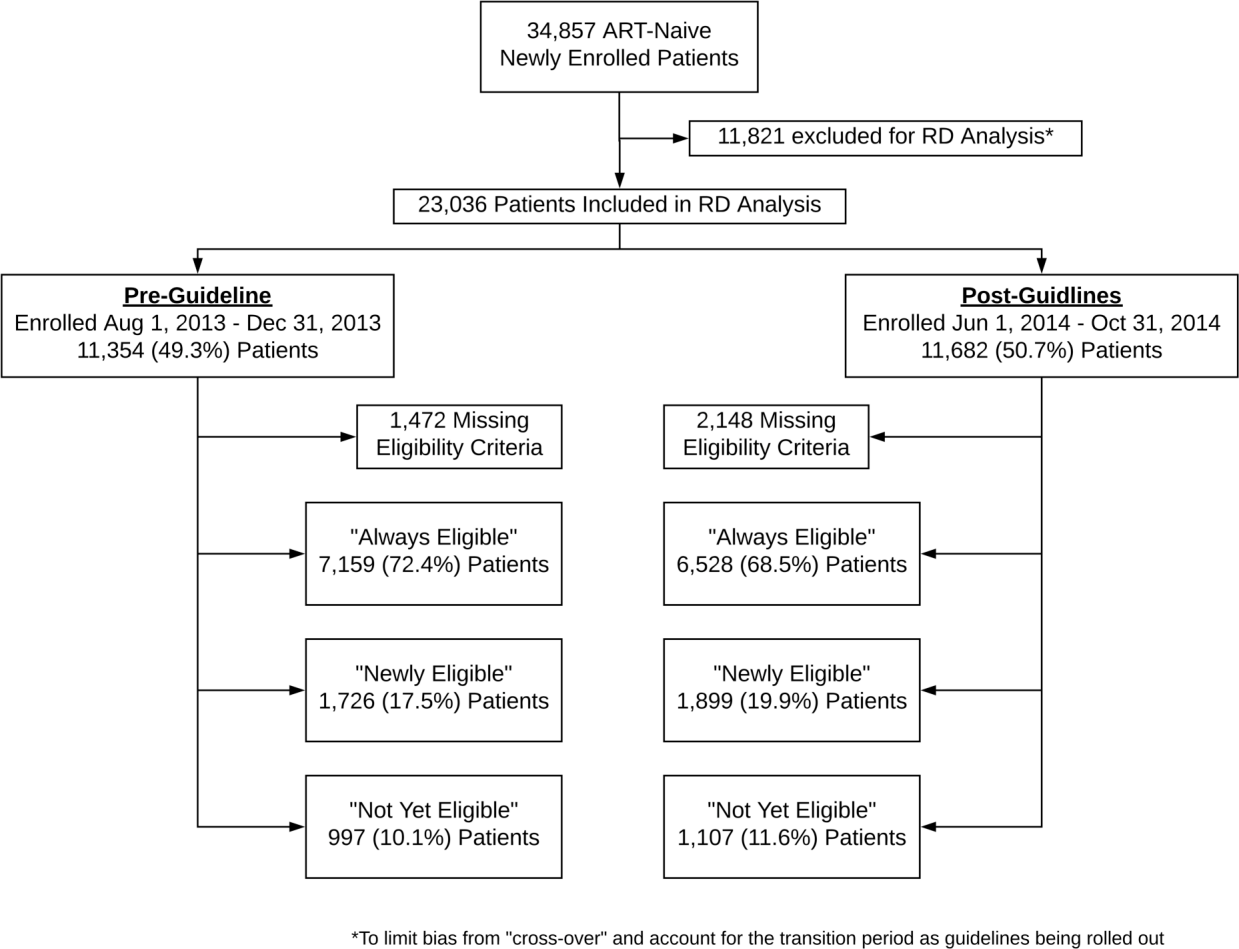
Patient flowchart.

Between August 1, 2013, and November 1, 2014, 34,857 ART-naïve patients newly enrolled at one of 64 ART clinics in four provinces in Zambia (Fig 1). Most were female (13,635 [39.1%] male, 13,998 [40.2%] not pregnant or breastfeeding female, 7,224 [20.7%] pregnant or breastfeeding female), with a median age of 34 years (IQR 28–41) and median CD4 count at enrollment of 268 cells/μL (IQR 134–430). At enrollment, 20,819 (70.1%) would have been eligible for ART by the 2010 guidelines, 5,578 (18.8%) would have been newly eligible under the expanded 2014 guidelines, and 3,319 (11.2%) would not have been considered eligible by either the 2010 or 2014 guidelines; 5,141 (14.8%) had unknown eligibility at enrollment due to missing CD4 counts (Table 1). Of newly eligible patients, 3,265 (58.5%) would have been eligible due to increasing the CD4 threshold only, and 2,313 (41.5%) would have become eligible due to implementation of Option B+ and being pregnant.

**Table 1:** Baseline patient characteristics by guideline exposure and eligibility group, *n* = 34,857.

Characteristics	All Patients		Always Eligible		Newly Eligible		Not Yet Eligible	
	Pre-guidelines (*n* = 18,584)	Post-guidelines (*n* = 16,273)	Pre-guidelines (*n* = 11,552)	Post-guidelines (*n* = 9,267)	Pre-guidelines (*n* = 2,880)	Post-guidelines (*n* = 2,698)	Pre-guidelines (*n* = 1,717)	Post-guidelines (*n* = 1,602)
Sex, *n* (%)								
Male	7,351 (39.6%)	6,284 (38.6%)	5,136 (44.5%)	4,187 (45.2%)	712 (24.7%)	670 (24.8%)	592 (34.5%)	552 (34.5%)
Non-pregnant female	7,529 (40.5%)	6,469 (39.8%)	4,340 (37.6%)	3,501 (37.8%)	1,003 (34.8%)	880 (32.6%)	1,125 (65.5%)	1,050 (65.5%)
Pregnant/breastfeeding female	3,704 (19.9%)	3,520 (21.6%)	2,076 (18.0%)	1,579 (17.0%)	1,165 (40.5%)	1,148 (42.6%)	0	0
Median age, years (IQR)	34 (28–41)	34 (28–41)	35 (29–41)	35 (30–42)	31 (26–38)	31 (26–37)	32 (27–39)	33 (27–40)
Median CD4 count, cells/μL (IQR)	264 (132–420)	272 (138–440)	185 (93–279)	182 (94–272)	435 (390–487)	438 (394–492)	627 (556–760)	631 (558–763)
WHO stage, *n* (%)								
I	8,638 (54.2%)	7,799 (57.1%)	4,433 (42.6%)	3,525 (43.0%)	1,893 (80.2%)	1,736 (78.9%)	1,125 (77.6%)	1,105 (82.5%)
II	3,136 (19.7%)	2,668 (19.5%)	1,817 (17.5%)	1,491 (18.2%)	468 (19.8%)	465 (21.1%)	324 (22.4%)	235 (17.5%)
III	3,808 (23.9%)	2,940 (21.5%)	3,808 (36.6%)	2,940 (35.8%)	0	0	0	0
IV	353 (2.2%)	251 (1.8%)	353 (3.4%)	251 (3.1%)	0	0	0	0
TB in past year, *n* (%)	860 (4.6%)	727 (4.5%)	860 (7.4%)	727 (7.8%)	0	0	0	0
Eligibility subgroup								
Always Eligible	11,552 (71.5%)	9,267 (68.3%)	-	-	-	-	-	-
Newly Eligible	2,880 (17.8%)	2,698 (19.9%)	-	-	-	-	-	-
Not Yet Eligible	1,717 (10.6%)	1,602 (11.8%)	-	-	-	-	-	-
Province								
Lusaka	3,204 (17.2%)	2,997 (18.4%)	1,895 (16.4%)	1,643 (17.7%)	516 (17.9%)	528 (19.6%)	364 (21.2%)	365 (22.8%)
Eastern	9,941 (53.5%)	8,591 (52.8%)	6,806 (58.9%)	5,286 (57.0%)	1,686 (58.5%)	1,540 (57.1%)	944 (55.0%)	871 (54.4%)
Southern	2,603 (14.0%)	2,290 (14.1%)	1,370 (11.9%)	1,090 (11.8%)	378 (13.1%)	333 (12.3%)	197 (11.5%)	232 (14.5%)
Western	2,836 (15.3%)	2,395 (14.7%)	1,481 (12.8%)	1,248 (13.5%)	300 (10.4%)	297 (11.0%)	212 (12.3%)	134 (8.4%)
Education								
None	1,145 (7.4%)	1,019 (7.7%)	707 (7.2%)	589 (7.6%)	163 (6.9%)	177 (8.0%)	81 (5.7%)	94 (7.2%)
Lower-mid basic	5,761 (37.5%)	4,728 (35.8%)	3,669 (37.5%)	2,791 (35.8%)	866 (36.5%)	760 (34.5%)	563 (39.6%)	495 (37.8%)
Upper basic/secondary	7,769 (50.5%)	6,873 (52.0%)	4,961 (50.7%)	4,021 (51.6%)	1,228 (51.7%)	1,180 (53.6%)	717 (50.5%)	673 (51.3%)
College/university	699 (4.5%)	597 (4.5%)	448 (4.6%)	389 (5.0%)	116 (4.9%)	86 (3.9%)	60 (4.2%)	49 (3.7%)
Marital status								
Single	2,097 (13.7%)	1,736 (13.2%)	1,306 (13.5%)	996 (13.0%)	306 (13.2%)	283 (12.8%)	193 (13.7%)	171 (13.4%)
Married	9,733 (63.8%)	8,496 (64.8%)	6,005 (62.1%)	4,771 (62.4%)	1,604 (69.4%)	1,578 (71.2%)	896 (63.5%)	818 (64.2%)
Divorced	2,087 (13.7%)	1,815 (13.8%)	1,421 (14.7%)	1,183 (15.5%)	248 (10.7%)	235 (10.6%)	200 (14.2%)	172 (13.5%)
Widowed	1,340 (8.8%)	1,068 (8.1%)	936 (9.7%)	697 (9.1%)	154 (6.7%)	121 (5.5%)	122 (8.6%)	114 (8.9%)
Disclosed HIV status	16,565 (97.2%)	14,354 (96.4%)	10,489 (97.3%)	8,345 (96.7%)	2,534 (97.2%)	2,387 (96.3%)	1,510 (97.9%)	1,395 (96.2%)

Abbreviations: TB, tuberculosis; WHO, World Health Organization.

### Longitudinal care experience from multistate analysis

**Fig 2:**
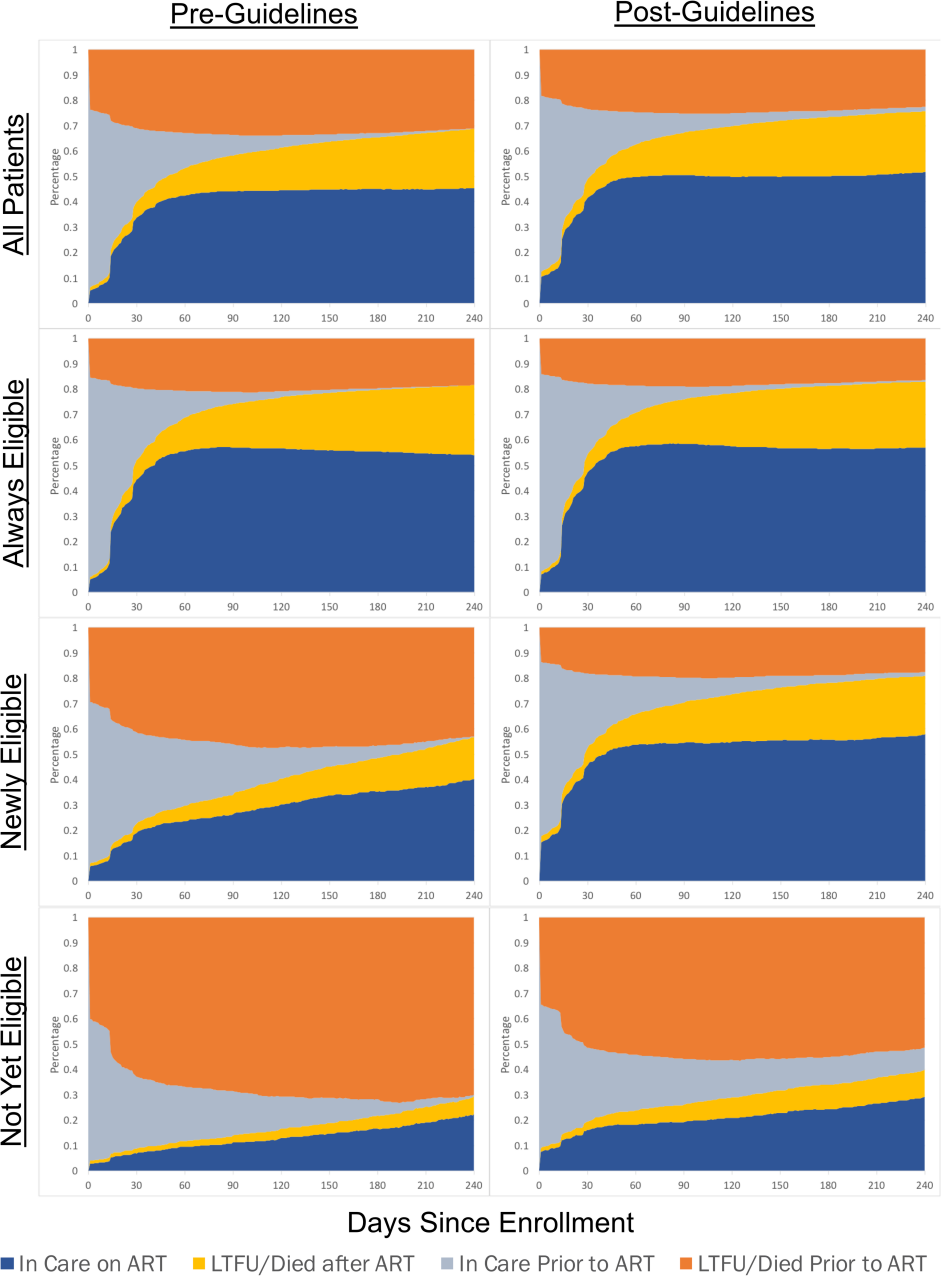
Multistate analysis of the longitudinal care experience of patients newly enrolling in care.

Overall, 73.5% (95% CI, 72.0%–74.9%) of all patients who enrolled after the guideline change had initiated ART by 180 days (compared to 65.5% [95% CI, 64.2%–66.8%] of patients prior to the guidelines) with only 2.5% (95% CI, 0.4%–4.6%) of patients still in care and not yet initiated at 180 days (compared to 1.6% [95% CI, 0.0%–3.6%] prior to the guidelines) (Fig 2). Still, only 50.1% (95% CI, 49.4%–50.9%) of patients were both in care and on ART at 180 days in the post-guideline period (compared to 45.1% prior to the guidelines [95% CI, 44.4%–45.8%]) with 47.4% of patients (95% CI, 46.1%–48.7%) either LTFU or dead at 180 days (compared to 53.3% in the pre-guideline period [95% CI, 52.0%–54.6%]). Among those LTFU or dead prior to initiating ART, a majority were lost after only one visit. When stratified by eligibility subgroups, these trends were most notable in the patients who were newly eligible for ART with the guideline change. In these newly eligible patients, there was an increase in patients both in care and on ART at 180 days (35.3% pre-guideline versus 55.7% post-guideline), but there was also a notable shift in LTFU or death from pre- to post-ART initiation for a substantial proportion of patients (13.2% LTFU or dead after initiating ART pre-guideline versus 22.5% post-guideline) (Fig 2).

### Effect of the implementation of new guidelines on ART initiation and retention in care

After restriction, 23,036 patients remained and were included in the regression discontinuity analysis. Patients were very similar when comparing those enrolling prior to implementation of the new guidelines to those presenting after the guidelines (both overall and across eligibility groups), as well as when comparing those included and excluded from the regression discontinuity analysis (Table 1, S1 Table). There were stable and continuous trends in the numbers of new patients enrolling and the total number of overall clinic visits in the pre- versus post-guideline period (S1 Fig).

**Fig 3:**
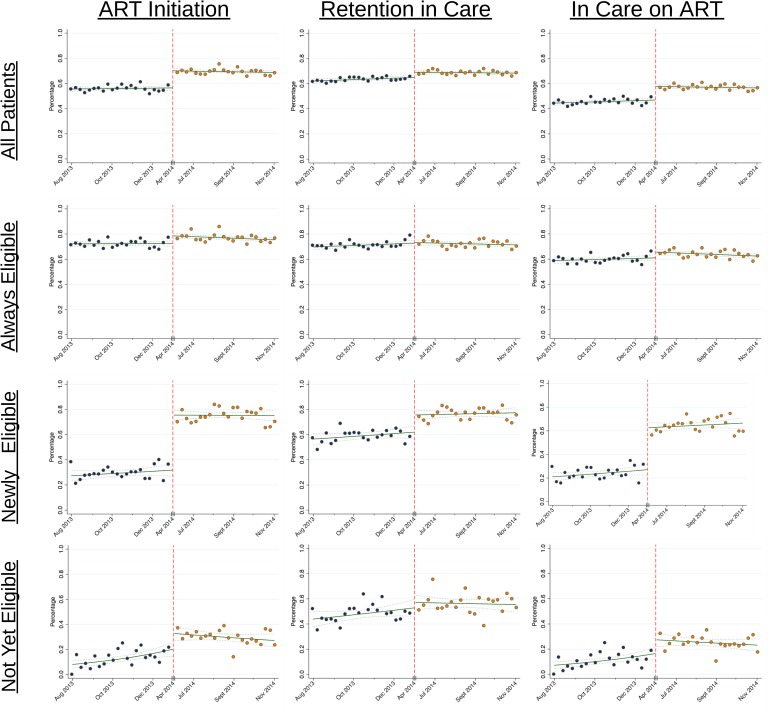
Results from regression discontinuity analyses on the effects of expanding treatment eligibility to patients with CD4 between 350 and 500 cells/μL and pregnant/breastfeeding women on ART initiation at 3 months, retention in care at 6 months, and the percentage of patients in care and on ART at 6 months.

There was an overall improvement in both ART initiation and retention in care in all newly enrolling ART-naïve patients in response to implementation of new treatment guidelines (Fig 3). Overall, implementation of new guidelines was associated with a 13.6% absolute increase in ART initiation by 3 months (95% CI, 11.1%–16.2%, *p* < 0.0001), a 4.1% absolute increase in retention in care at 6 months (95% CI, 1.6%–6.7%, *p* = 0.0014), and a 10.8% absolute increase in the percentage of patients who initiated ART by 3 months and were still in care at 6 months (95% CI, 8.1%–13.5%, *p* < 0.0001) (Table 2 and Fig 3). All results were robust to model specification in sensitivity analyses and were similar when stratified by sex (S2 Table, S3 Table).

**Table 2:** Results of regression discontinuity analysis on the effects of implementing new HIV treatment guidelines on ART initiation and loss to follow-up*, *n* = 23,036.

Outcome	Pre-Guidelines		Post-Guidelines		Risk Difference		*p*-value
	Percent	95% CI	Percent	95% CI	Percent	95% CI	
**All Patients**							
ART initiation	56.4	54.5–58.4	70.1	68.4–71.8	13.6	11.1–16.2	<0.0001
Retention in care	64.9	63.0–66.8	69.1	67.3–70.8	4.1	1.6–6.7	0.0014
In care on ART	46.9	45.0–48.9	57.7	55.9–59.6	10.8	8.1–13.5	<0.0001
**Always Eligible**							
ART initiation	72.4	70.2–74.6	78.5	76.5–80.6	6.2	3.2–9.2	0.0001
Retention in care	72.8	70.5–75.0	73.2	71.0–75.4	0.4	−2.7–3.6	0.79
In care on ART	61.0	58.6–63.4	65.6	63.3–68.0	4.6	1.2–8.0	0.008
**Newly Eligible**							
ART initiation	31.8	27.1–36.5	75.5	71.4–79.5	43.7	37.5–49.9	<0.0001
Retention in care	62.0	57.0–66.9	75.6	71.7–79.6	13.6	7.3–20.0	<0.0001
In care on ART	27.1	22.5–31.7	62.6	58.2–67.0	35.5	29.2–41.9	<0.0001
**Not Yet Eligible**							
ART initiation	20.2	14.5–25.9	32.7	27.0–38.5	12.5	4.5–20.6	0.002
Retention in care	52.9	46.2–59.5	57.1	51.2–63.0	4.3	−4.6–13.2	0.35
In care on ART	16.6	11.5–21.6	27.4	22.0–32.8	10.8	3.4–18.3	0.004

*Estimates derived using a modified Poisson regression with robust variances.

Abbreviation: ART, antiretroviral therapy.

The most significant improvements were seen in patients who became newly eligible for ART with the 2014 update to the guidelines (Fig 3). These patients had a 43.7% increase in ART initiation (95% CI, 37.5%–49.9%, *p* < 0.0001), a 13.6% increase in retention (95% CI, 7.3%–20.0%, *p* < 0.0001), and a 35.5% increase in the percentage of patients who were in care and on ART at 6 months (95% CI, 29.2%–41.9%, *p* < 0.0001) (Table 2). The effect of implementing new guidelines was similar for those patients who were pregnant and became newly eligible under Option B+ compared to both females and males who became newly eligible because of CD4 count only (Table S5).

For those patients who would not yet have been eligible by either the 2010 or 2014 guidelines as well as those who were always eligible during our study period based on the 2010 guidelines, instituting new guidelines increased ART initiation by 3 months (“Not Yet Eligible” RD +12.5% [95% CI, 4.5%–20.6%, *p* = 0.0023]; “Always Eligible” RD + 6.2% [95% CI, 3.2%–9.2%, *p* = 0.0001]) and the percentage of patients in care and on ART at 6 months (“Not Yet Eligible” RD + 10.8% [95% CI, 3.4%–18.3%, *p* = 0.0042]; “Always Eligible” RD + 4.6% [95% CI, 1.2%–8.0%, *p* = 0.0076]) but had a limited effect on retention in care (Table 2 and Fig 3).

### Effect of ART initiation on retention in care

Using the implementation of new guidelines as an IV for ART initiation and assuming the underlying IV assumptions were met, we estimated the causal effect of initiating ART in response to the guideline change on subsequent retention in care. In the overall population, initiating ART because of the guideline change led to a 37.9% (95% CI, 28.8%–46.9%, *p* < 0.0001) increase in retention in care; the effect was similar when restricted only to asymptomatic patients with a CD4 count above 350 cells/μL. This result indicates that 2.6 patients (95% CI, 2.1–3.5) would need to be started on ART to prevent one from becoming LTFU (Table 3).

**Table 3:** Results of instrumental variable analysis on the effect of ART initiation on retention in care*, *n* = 23,036.

Measure	Estimate	95% CI	*p*-value
**Risk difference, %**	37.9	28.8–46.9	<0.0001
**Number needed to treat**	2.6	2.1–3.5	

*Estimates derived using two-stage least squares bivariate probit regression.

Abbreviation: ART, antiretroviral therapy.

## Discussion

Using a regression discontinuity design, we found that guidelines expanding ART eligibility improved ART initiation and retention in care in a large network of facilities in Zambia. These guidelines, which increased the CD4 threshold for treatment from 350 to 500 cells/μL and included all pregnant women, were associated with a 13.6% absolute increase in ART initiation within 3 months, a 4.1% increase in retention in care at 6 months, and a 10.8% increase in the percentage of newly enrolling ART-naïve patients on ART and still in care at 6 months. These effects were greatest in patients who became newly eligible for ART with the change in guidelines, with a 43.7% increase in ART initiation, a 13.6% increase in retention, and a 35.5% increase in the percentage of patients on ART and still in care at 6 months. Furthermore, we show that actually initiating ART led to a 37.9% increase in retention in care at 6 months; translated into a number needed to treat, this indicates that one episode of LTFU was averted for every 2.6 people initiated on ART because of these guideline changes. These results were robust to sensitivity analyses that varied model specifications, and, to the extent that this analysis meets the underlying assumptions of regression discontinuity and IV analyses, these findings indicate that expanding ART eligibility improves engagement in the population overall.

The experience of expanding the treatment threshold from a CD4 of 350 to 500 cells/μL could foreshadow the effects of further ART expansion to universal treatment, which is presently being implemented globally. Previous studies from South Africa—including a recent analysis using a regression discontinuity design comparing patients presenting just above and below a CD4 treatment threshold of 350 cells/μL—suggest that offering treatment enhances both ART initiation and retention in care [10,26,34–37]. Our work confirms and extends this observation using a different discontinuity: by examining patients immediately before and after a guideline change, our estimates reflect changes induced in the behavior of health systems as well as patients because of the guidelines. Despite ongoing concerns that asymptomatic patients with higher CD4 counts may be less engaged in HIV care due to the lack of immediately apparent benefits of treatment, our data suggest that they will have similar rates of ART initiation and retention in care if eligible for ART, as compared to their more symptomatic counterparts [38–44]. This speaks directly to the powerful behavioral effects of initiating ART, regardless of CD4 count, in addition to the biologic effects measured in HPTN 052, INSIGHT START, and TEMPRANO [1–3]. As the major determinants of HIV-related health behaviors are likely to be clinical status and the presence of symptoms rather than the CD4 count itself, it is likely that the perceptions of HIV care do not markedly differ between treatment-eligible patients with a CD4 between 350 and 500 cells/μL and those with a CD4 above 500 cells/μL. Although our estimates are specific to the 2014 guideline change in Zambia, they are thus conceptually very similar to the effects of a guideline change to remove the CD4 threshold for treatment altogether.

Although retention after ART initiation was similar in symptomatic and asymptomatic patients, the mechanisms behind the behavioral effects of initiating ART are multifactorial and may differ between these subsets of patients. Across CD4 counts, improved retention after ART initiation may reflect an increase in perceived “cost” associated with stopping ART after being initiated as compared to simply never starting (i.e., loss aversion), the persisting benefit of counseling and disclosure that occurs in preparation for ART initiation, or the additional services that patients receive once they have started on ART [10]. Additionally, those who present to care earlier, when their CD4 count is high, may also have other motivating factors apart from symptoms, such as increased health-seeking behaviors leading to earlier diagnosis, increasing knowledge of the long-term benefits of ART in the community, or personally witnessing the devastating effects of advanced HIV disease. Further efforts to enhance retention should seek to better understand and leverage these different behavioral mechanisms to improve patient engagement.

It is important to note that we also did not observe any negative spillover effects associated with expanding ART eligibility. In fact, we noted a slight increase in ART initiation (RD + 6.2%) without any changes to retention in the patients who were eligible for ART even prior to the guideline change, which may have been due to an overall expansion of the ART supply. We also saw increased ART initiation in those who were not yet eligible for ART (RD +12.5%). We posit that this may have also been related to an informal loosening of eligibility criteria for those patients not yet officially eligible for ART, as there were no other changes made to the guidelines at that time that would have been expected to affect patient care in such a way [21]. Lastly, we did not observe a trend towards an increased number of overall clinic visits despite the increase in the number of patients on ART and in care. This is likely due to the concurrent trend in increasing the appointment intervals for patients who have been on ART for more than 6 months and are considered “stable,” which would not have affected the care received by newly enrolling patients but would have offset the increased visit burden. Thus, despite concern that expanding ART eligibility could potentially lead to crowding out of sicker patients and increased clinic congestion, we observed overall increases in ART initiation and retention in care without such associated negative spillover effects [19].

Despite the overall benefits of expanding ART eligibility, with more patients initiated on treatment and retained in care, significant gaps are likely to remain in the care cascade even with adoption of treat-all. First of all, in our population, about 70% of patients still presented with more advanced disease and would have been eligible by the 2010 guidelines, highlighting the need for stronger efforts to diagnose patients and link them to care earlier. Furthermore, despite enrolling in care and being ART eligible, a significant proportion of patients still never initiate ART; a majority of these patients are LTFU after only one visit. Among those who do start ART, there is an additional subset that becomes LTFU shortly afterward, for whom the effect of expanding eligibility is in essence simply to shift attrition from pre-ART initiation to post-ART initiation. Currently, there is little evidence as to how this shift in attrition from pre- to post-ART initiation—also seen in the same-day ART initiation studies—affects subsequent re-engagement and drug resistance [36,45,46]. Thus, as the world transitions to treat-all, there will remain an urgent need for HIV care programs to address remaining gaps in the care cascade by developing targeted tools to transition towards more comprehensive and patient-centered care [47,48].

Regression discontinuity and IV study designs can, provided that the central underlying assumptions can be met, quantify both the intended and unintended effects of interventions implemented in routine care and are thus a powerful, though underused, tool in the implementation science armamentarium. Traditional RCTs have high internal validity, but their external generalizability—imperative for informing policy decisions—is often limited [7–9]. For example, in contrast to our findings, there were no differences in retention in RCTs assessing early versus delayed ART by design, but this also probably leads to an underestimate of the true benefit of initiating ART in the real world, where the benefits are likely both biological (i.e., viral suppression) and behavioral (i.e., retention). Furthermore, these designs are able to identify spillover effects such as those seen in our study but could also be used to identify effects on HIV testing, stigma, transmission, and socioeconomic status [11–19]. Expanding the use of regression discontinuity and IV designs in public health research thus offers a solution with both high internal and external validity and can also be used for settings where RCTs are not feasible for ethical, economical, or political reasons [8,9,27].

There are several limitations of our study. First, it is important to recognize that our results, strictly speaking, are only applicable to the current care setting, under the core assumptions of the regression discontinuity and IV analyses. This makes generalizability to populations that are diagnosed, linked to care, or initiated on ART under different circumstances—such as with home-based testing, enhanced linkage procedures, same-day ART initiation, or hospitalization—uncertain. As we only assessed patients already presenting to clinic, we were also unable to assess whether expanding eligibility had more global effects, such as on patients’ decision to go for HIV testing or overall rates of linkage to care, although the similar number of patients enrolling in care prior to and after the guideline change would suggest minimal effects on earlier stages of the HIV care cascade. Second, we excluded patients enrolling closest to the implementation date to minimize bias both from “cross over” and also the transition period during gradual guideline rollout, precluding us from comparing patients enrolling immediately before and after the guideline change. Nevertheless, calendar time was not a significant predictor in our models, making it unlikely that secular trends over this time period meaningfully affected our results in the context of our patient restriction. Additionally, our results remained robust to sensitivity analyses varying criteria for patient restriction and also when adjusted for baseline patient covariates, despite a slight change in patient composition before and after the guidelines. Third, we were unable to assess virologic outcomes, as viral loads were not routinely collected in Zambia during our study period. Still, recent results from the Zambia Population-Based HIV Impact Assessment (ZAMPHIA) suggest that just under 90% of patients in care and on ART will be virally suppressed [49]. Lastly, there were inherent limitations in our data source that could have led to misclassified eligibility in the stratified analysis. This could have led us to misidentify effects in patients not directly targeted by the guidelines, although model diagnostics, including sensitivity analyses and falsification tests, suggested that any potential bias was negligible.

In conclusion, we found that the 2014 change in Zambia’s HIV treatment guidelines was associated with increased ART initiation, retention in care, and the percentage of patients on ART and still in care at 6 months without evidence of associated negative spillover effects, providing important new data on the effects of expanding ART eligibility to asymptomatic patients in sub-Saharan Africa. Furthermore, we quantify the behavioral effect of actually initiating ART on retention in care and also show that a substantial subset of patients will not see the benefits of ART despite being eligible for treatment, largely due to attrition. Although these results are highly relevant to Zambia’s transition to universal treatment in December 2016, further study is still needed into the actual experience of treat-all—particularly with regard to viral suppression and in the setting of new care models meant to improve the HIV care cascade [50]. Moreover, as the questions about the “who” and “when” of ART initiation are coming to an end, we still need a better understanding of those patients who do poorly despite being eligible for treatment and to implement interventions to expand HIV testing, keep people engaged in care, and differentially target resources according to patient needs and preferences [44].

## Supporting information

S1 STROBE Checklist STROBE checklist. (DOCX)

S1 Appendix Methods for verifying the underlying assumptions of the instrumental variable analysis. (DOCX)

S1 Fig Trends in new enrollees and daily clinic visits. (TIF)

S1 Table Baseline patient characteristics by enrollment date. (DOCX)

S2 Table Results of regression discontinuity sensitivity analyses. (DOCX)

S3 Table Results of regression discontinuity analysis, stratified by sex. (DOCX)

## Abbreviations

ART: antiretroviral therapy
CIDRZ: Centre for Infectious Disease Research in Zambia
HBV: hepatitis B virus
IV: instrumental variable
LATE: local average treatment effect
LTFU: lost to follow-up
PLWH: person living with HIV
RCT: randomized controlled trial
RD: risk difference
WHO: World Health Organization
UNZABREC: University of Zambia Biomedical Research Ethics Committee
ZAMPHIA: Zambia Population-Based HIV Impact Assessment
