# Supplementary material for: Estimating the real-world effects of expanding antiretroviral treatment eligibility: Evidence from a regression discontinuity analysis in Zambia
Source: PLoS Med. 2018 Jun 5;15(6):e1002574. doi: 10.1371/journal.pmed.1002574 (PMC5988277; doi:10.1371/journal.pmed.1002574)
Supplement: S1 Appendix — (DOCX) [file pmed.1002574.s002.docx]

**S1 Appendix – Methods for verifying the underlying assumptions of the instrumental variable analysis**

We estimated the local average treatment effect (LATE) of ART initiation on retention in care in response by performing an instrumental variable (IV) analysis using exposure to the guidelines as the IV, ART initiation as the treatment, and retention in care as the outcome of interest. Prior to conducted this analysis, we first assessed for violations of the underlying IV assumptions to verify the appropriateness of performing an this analysis. These assumptions are 1) the IV is associated with treatment, 2) the IV is not associated with the outcome except through its effect on the treatment, 3) there are no common causes (i.e. unmeasured confounders) of the IV and the outcome, and 4) exposure to the IV does not lead to anyone being untreated when they otherwise would have been [1–3]. The first assumption—that the IV is associated with the treatment—was empirically verified by the primary regression discontinuity analysis that showed the change in guidelines led to increases in ART initiation. The second assumption—that the IV is associated with the outcome only through its effect on treatment—was assessed by performing a logistic regression with exposure to the guidelines (the IV) and ART initiation (the treatment) as independent variables and retention as the outcome; we then confirmed that there was no evidence that exposure to the guidelines was associated with retention when adjusting for ART initiation. We were unable to empirically assess the third assumption—that there is no unmeasured confounding between the IV and outcome—or the fourth assumption—that exposure to the IV does not lead to anyone being untreated when they otherwise would have been—but felt these were valid based on a priori hypothesis of the causal relationships between the new guidelines, ART initiation, and retention in care and the fact that there were no additional policies rolled out concurrently with expanded ART eligibility that would be expected to impact retention [4,5].

**References**

1. Hernan MA, Robins JM. Instruments for causal inference: an epidemiologist's dream? Epidemiology. 2006;17(4):360-72.

2. Swanson SA, Hernan MA. Commentary: how to report instrumental variable analyses (suggestions welcome). Epidemiology. 2013;24(3):370-4.

3. Barnighausen T, Oldenburg C, Tugwell P, Bommer C, Cara E, Barreto M, et al. Quasi-experimental study designs series - Paper 7: assessing the assumptions. J Clin Epidemiol. 2017;89:53–66.

4. Zambia Adult and Adolescent Antiretroviral Therapy Protocols. 2010. Available from: <http://www.who.int/hiv/pub/guidelines/zambia_art.pdf>.

5. Zambia Consolidated Guidelines for Treatment and Prevention of HIV Infection. 2014. Available from: <http://www.moh.gov.zm/docs/reports/Consolidated%20Guidelines%20Final%20Feb%202014.pdf>.
