# Supplementary figures and images for: Estimating the real-world effects of expanding antiretroviral treatment eligibility: Evidence from a regression discontinuity analysis in Zambia
Source: PLoS Med. 2018 Jun 5;15(6):e1002574. doi: 10.1371/journal.pmed.1002574 (PMC5988277; doi:10.1371/journal.pmed.1002574)

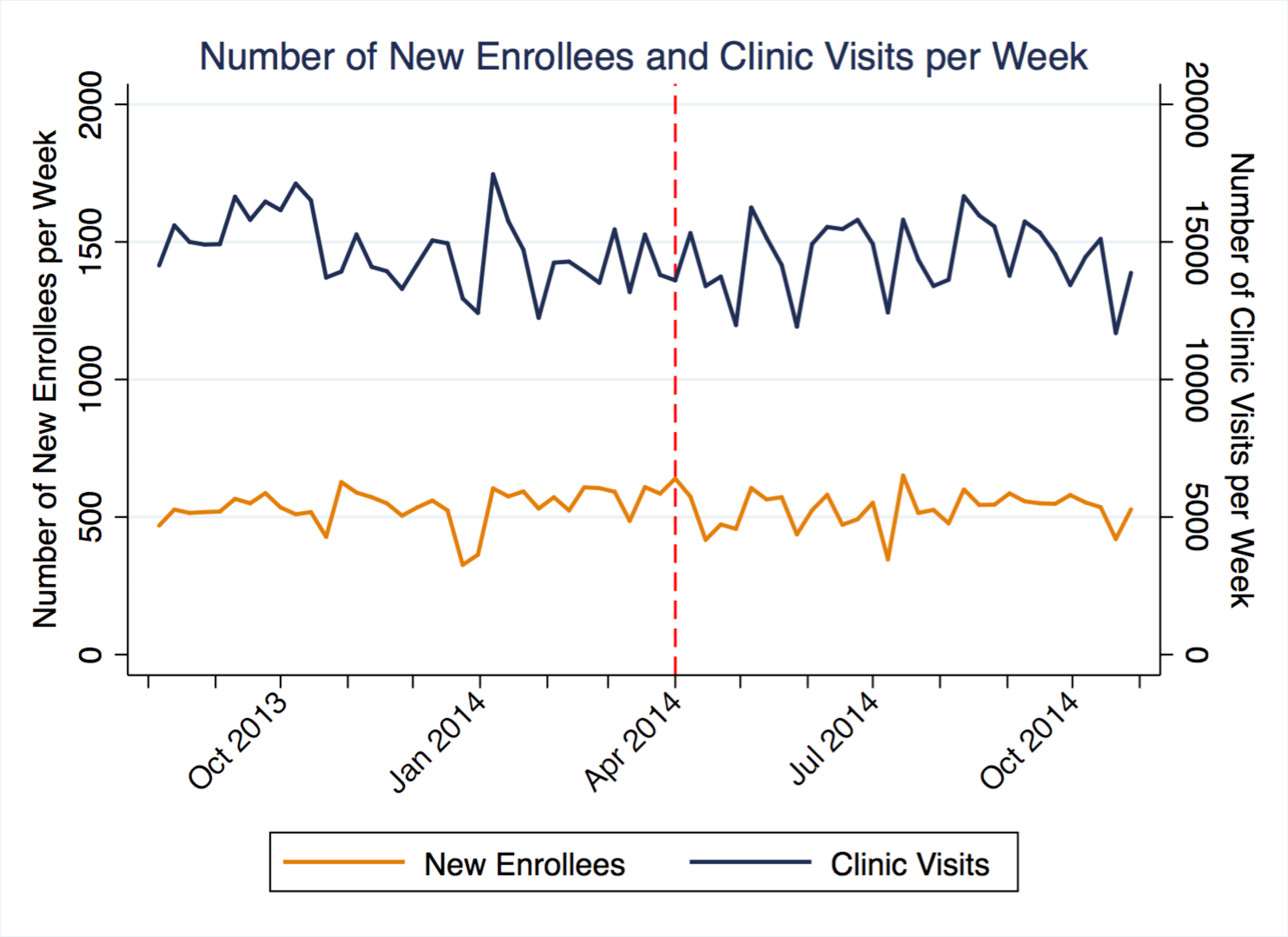

Supplement: S1 Fig — (TIF) [file pmed.1002574.s003.tif]
