# Supplementary material for: Estimating the real-world effects of expanding antiretroviral treatment eligibility: Evidence from a regression discontinuity analysis in Zambia
Source: PLoS Med. 2018 Jun 5;15(6):e1002574. doi: 10.1371/journal.pmed.1002574 (PMC5988277; doi:10.1371/journal.pmed.1002574)
Supplement: S1 Table — (DOCX) [file pmed.1002574.s004.docx]

**S1 Table: Baseline patient characteristics by enrollment date, n=34,857**

| Baseline Patient Characteristics by Enrollment Date, n=34,857 | | | |
| --- | --- | --- | --- |
|  | **Enrollment Date** | | |
|  | **Aug 1, 2013 –**  **Dec 31, 2013**  **(n=11,345)** | **Jan 1, 2014 –**  **May 31, 2014**  **(n=11,821)** | **Jun 1, 2014 –**  **Oct 31, 2014**  **(n=11,682)** |
| Sex, n (%) |  |  |  |
| Male | 4,443 (39.1%) | 4,743 (40.1%) | 4,449 (38.1%) |
| Non-pregnant female | 4,565 (40.2%) | 4,766 (40.3%) | 4,667 (40.0%) |
| Pregnant/breastfeeding female | 2,346 (20.7%) | 2,312 (19.6%) | 2,566 (22.0%) |
| Median age, years (IQR) | 34 (28, 41) | 34 (28, 40) | 34 (28, 41) |
| Median CD4 count, cells/μL (IQR) | 258 (130, 413) | 274 (138, 434) | 271 (137, 440) |
| WHO stage, n (%) |  |  |  |
| I | 5,251 (54.0%) | 5,607 (55.5%) | 5,579 (57.2%) |
| II | 1,895 (19.5%) | 1,976 (19.5%) | 1,933 (19.8%) |
| III | 2,343 (24.1%) | 2,336 (23.1%) | 2,069 (21.2%) |
| IV | 234 (2.4%) | 191 (1.9%) | 179 (1.8%) |
| TB in past 6 months, n (%) | 570 (5.0%) | 483 (4.1%) | 534 (4.6%) |
| Eligibility subgroup |  |  |  |
| Always eligible | 7,159 (72.4%) | 7,132 (69.2%) | 6,528 (68.5%) |
| Newly eligible | 1,726 (17.5%) | 1,953 (19.0%) | 1,899 (19.9%) |
| Not yet eligible | 997 (10.1%) | 1,215 (11.8%) | 1,107 (11.6%) |
| Province |  |  |  |
| Lusaka | 2,050 (18.1%) | 1,894 (16.0%) | 2,257 (19.3%) |
| Eastern | 6,012 (53.0%) | 6,410 (54.2%) | 6,110 (52.3%) |
| Southern | 1,596 (14.1%) | 1,665 (14.1%) | 1,632 (14.0%) |
| Western | 1,696 (14.9%) | 1,852 (15.7%) | 1,683 (14.4%) |
| Education |  |  |  |
| None | 721 (7.7%) | 719 (7.4%) | 724 (7.7%) |
| Lower-mid basic | 3,536 (37.6%) | 3,577 (36.6%) | 3,376 (35.9%) |
| Upper basic/secondary | 4,724 (50.2%) | 5,027 (51.4%) | 4,891 (52.0%) |
| College/university | 428 (4.5%) | 450 (4.6%) | 418 (4.4%) |
| Marital status |  |  |  |
| Single | 1243 (13.3%) | 1,363 (14.1%) | 1,227 (13.1%) |
| Married | 5,973 (64.0%) | 6,175 (63.9%) | 6,081 (64.8%) |
| Divorced | 1,284 (13.8%) | 1,326 (13.7%) | 1,292 (13.8%) |
| Widowed | 834 (8.9%) | 796 (8.2%) | 778 (8.3%) |
| Disclosed HIV Ssatus | 10,207 (97.1%) | 10,478 (97.1%) | 10,234 (96.3%) |
