# Supplementary material for: Estimating the real-world effects of expanding antiretroviral treatment eligibility: Evidence from a regression discontinuity analysis in Zambia
Source: PLoS Med. 2018 Jun 5;15(6):e1002574. doi: 10.1371/journal.pmed.1002574 (PMC5988277; doi:10.1371/journal.pmed.1002574)
Supplement: S2 Table — (DOCX) [file pmed.1002574.s005.docx]

**S2 Table: Results of regression discontinuity sensitivity analyses**

| Results of Regression Discontinuity Sensitivity Analyses* | | | | | | | | | | | | |
| --- | --- | --- | --- | --- | --- | --- | --- | --- | --- | --- | --- | --- |
|  | **Final Model^a^** | | **IK Bandwidth^b^** | | **50-day Bandwidth** | | **30-Day Transition Period^c^** | | **Adjusted^d^** | | **Weighted^e^** | |
|  | Risk Difference | 95% CI | Risk Difference | 95% CI | Risk Difference | 95% CI | Risk Difference | 95% CI | Risk Difference | 95% CI | Risk Difference | 95% CI |
| **All patients** |  |  |  |  |  |  |  |  |  |  |  |  |
| ART initiation | 13.6 | 11.1 – 16.2 | 13.7 | 10.7 - 16.7 | 16.1 | 11.4 – 20.7 | 11.1 | 8.6 – 13.5 | 14.6 | 12.0 – 17.3 | 13.6 | 10.8 – 16.4 |
| Retention in care | 4.1 | 1.6 – 6.7 | 5.4 | 1.4 - 9.3 | 4.9 | 0.3 – 9.6 | 3.4 | 0.9 – 5.8 | 3.8 | 1.1 – 6.5 | 4.5 | 1.7 – 7.3 |
| In care on ART | 10.8 | 8.1 – 13.5 | 10.8 | 6.8 - 14.9 | 12.3 | 7.5 – 17.1 | 8.9 | 6.3 – 11.4 | 12.0 | 9.1 – 14.9 | 10.6 | 7.7 – 13.6 |
|  |  |  |  |  |  |  |  |  |  |  |  |  |
| IV estimate | 37.9 | 28.8 – 46.9 | 33.3 | 19.1 - 47.5 | 38.3 | 21.4 – 55.2 | 38.6 | 29.5 – 47.7 | 35.5 | 26.4 – 44.7 | 35.1 | 24.7 – 45.5 |
|  |  |  |  |  |  |  |  |  |  |  |  |  |
| **Always eligible** |  |  |  |  |  |  |  |  |  |  |  |  |
| ART initiation | 6.2 | 3.2 – 9.2 | 5.5 | 1.2 - 9.8 | 8.3 | 2.9 – 13.7 | 4.9 | 2.0 – 7.7 | 5.9 | 2.9 – 8.9 | 6.0 | 2.7 – 9.3 |
| Retention in care | 0.4 | -2.7 – 3.6 | -0.2 | -5.0 - 4.7 | 0.3 | -5.4 – 5.9 | -0.3 | -3.3 – 2.7 | 0.4 | -2.7 – 3.5 | 0.1 | -3.3 – 3.5 |
| In care on ART | 4.6 | 1.2 – 8.0 | 4.3 | -0.1 - 8.8 | 6.5 | 0.4 – 12.7 | 3.7 | 0.5 – 6.9 | 4.5 | 1.1 – 7.9 | 4.1 | 0.4 – 7.8 |
|  |  |  |  |  |  |  |  |  |  |  |  |  |
| **Newly eligible** |  |  |  |  |  |  |  |  |  |  |  |  |
| ART initiation | 43.7 | 37.5 – 49.9 | 37.1 | 27.9 - 46.3 | 40.0 | 28.5 – 51.6 | 40.0 | 34.1 – 46.0 | 46.4 | 40.2 – 52.6 | 40.9 | 34.1 – 47.7 |
| Retention in care | 13.6 | 7.3 – 20.0 | 12.3 | 2.6 - 22.1 | 11.0 | -0.4 – 22.3 | 12.8 | 6.7 – 18.8 | 14.4 | 8.1 – 20.7 | 13.8 | 6.9 – 20.7 |
| In care on ART | 35.5 | 29.2 – 41.9 | 29.8 | 20.1 39.5 | 31.0 | 19.9 – 42.1 | 32.1 | 26.1 – 38.1 | 38.4 | 32.1 – 44.7 | 33.3 | 26.4 – 40.2 |
|  |  |  |  |  |  |  |  |  |  |  |  |  |
| **Not yet eligible** |  |  |  |  |  |  |  |  |  |  |  |  |
| ART initiation | 12.5 | 4.5 – 20.6 | 14.1 | 3.6 - 24.5 | 16.6 | 3.2 – 30.0 | 7.0 | -0.3 – 14.2 | 9.2 | 1.4 – 17.0 | 14.5 | 5.9 – 23.0 |
| Retention in care | 4.3 | -4.6 – 13.2 | 11.5 | 0 - 23.0 | 13.6 | -1.8 – 29.0 | 0.1 | -8.4 – 8.5 | 2.5 | -6.4 – 11.4 | 7.7 | -1.9 – 17.3 |
| In care on ART | 10.8 | 3.4 – 18.3 | 12.7 | 2.1 - 23.3 | 14.3 | 2.6 – 26.0 | 5.6 | -1.0 – 12.2 | 8.3 | 1.0 – 15.7 | 12.7 | 5.0 – 20.4 |

*Estimates derived using a modified Poisson regression with robust variances

^a^ Final model used 153 day bandwidth, 60 day transition period, was unadjusted, and used rectangular weighting kernel.

^b^ Bandwidths derived using Imbens-Kalyanaram algorithm to objectively attempt to identify the largest window around the cutoff where the relationship between time and the outcome are approximately linear

^c^ Transition period reduced to 30 days (i.e., only excluded patients enrolling 30 days after guideline enrollment)

^d^ Adjusted for age, sex, CD4 count at enrollment, and clinic

^e^ Observations weighted using a triangular-shaped kernel to assign greater weight proportionally to how close observations were to the threshold
