# Supplementary material for: Estimating the real-world effects of expanding antiretroviral treatment eligibility: Evidence from a regression discontinuity analysis in Zambia
Source: PLoS Med. 2018 Jun 5;15(6):e1002574. doi: 10.1371/journal.pmed.1002574 (PMC5988277; doi:10.1371/journal.pmed.1002574)
Supplement: S3 Table — (DOCX) [file pmed.1002574.s006.docx]

**S3 Table: Results of regression discontinuity analysis stratified by sex**

| Results of Regression Discontinuity Stratified by Sex* | | | | | | | | |
| --- | --- | --- | --- | --- | --- | --- | --- | --- |
|  | **Male** | |  | **Nonpregnant Female** | |  | **Pregnant Female** | |
|  | Risk Difference | 95% CI |  | Risk Difference | 95% CI |  | Risk Difference | 95% CI |
| **All patients** |  |  |  |  |  |  |  |  |
| ART initiation | 10.9 | 6.8 – 15.0 |  | 11.8 | 7.7 – 16.0 |  | 19.5 | 14.3 – 24.6 |
| Retention in care | 4.5 | 0.4 – 8.6 |  | 2.1 | -1.9 – 6.2 |  | 5.1 | -0.2 – 10.4 |
| In care on ART | 8.0 | 3.6 – 12.3 |  | 9.0 | 4.7 – 13.2 |  | 17.1 | 11.4 – 22.8 |
|  |  |  |  |  |  |  |  |  |
| IV estimate | 42.9 | 23.6 – 62.3 |  | 32.7 | 17.5 – 47.9 |  | 37.7 | 24.8 – 50.6 |
|  |  |  |  |  |  |  |  |  |
| **Always eligible** |  |  |  |  |  |  |  |  |
| ART initiation | 4.1 | -0.4 – 8.7 |  | 5.5 | 0.4 – 10.6 |  | 10.1 | 4.5 – 15.8 |
| Retention in care | 1.2 | -3.6 – 6.1 |  | -1.0 | -6.1 – 4.2 |  | -0.4 | -6.9 – 6.1 |
| In Ccre on ART | 3.3 | -1.9 – 8.5 |  | 3.0 | -2.6 – 8.6 |  | 9.0 | 1.8 – 16.2 |
|  |  |  |  |  |  |  |  |  |
| **Newly eligible** |  |  |  |  |  |  |  |  |
| ART initiation | 52.4 | 40.6 – 64.1 |  | 42.7 | 31.9 – 53.4 |  | 37.3 | 27.2 – 47.4 |
| Retention in care | 18.0 | 4.9 - 31.1 |  | 13.3 | 3.0 – 23.6 |  | 11.0 | 0.8 – 21.2 |
| In care on ART | 36.6 | 23.9 – 49.3 |  | 38.6 | 27.9 – 49.2 |  | 31.3 | 21.0 – 41.6 |
|  |  |  |  |  |  |  |  |  |
| **Not yet eligible** |  |  |  |  |  |  |  |  |
| ART initiation | 11.6 | -2.0 – 25.1 |  | 13.7 | 3.6 – 23.7 |  | - | - |
| Retention in care | 12.2 | -1.6 – 26.0 |  | -0.7 | -12.2 – 10.8 |  | - | - |
| In care on ART | 8.5 | -3.3 – 20.3 |  | 13.1 | 3.5 – 22.6 |  | - | - |

*Estimates derived using a modified Poisson regression with robust variances
